# Interdisciplinary Transgender Veteran Care: Development of a Core Curriculum for VHA Providers

**DOI:** 10.1089/trgh.2015.0004

**Published:** 2016-02-01

**Authors:** Jillian C. Shipherd, Michael R. Kauth, Anthony F. Firek, Ranya Garcia, Susan Mejia, Sandra Laski, Brent Walden, Sonia Perez-Padilla, Jan A. Lindsay, George Brown, Lisa Roybal, Colton L. Keo-Meier, Herschel Knapp, Laura Johnson, Rebecca L. Reese, William Byne

**Affiliations:** ^1^ Lesbian, Gay, Bisexual, and Transgender (LGBT) Program, Veterans Health Administration, Washington, District of Columbia.; ^2^ VA Boston Healthcare System, Boston, Massachusetts.; ^3^ Women's Health Sciences Division, National Center for PTSD, Boston, Massachusetts.; ^4^ Boston University School of Medicine, Boston, Massachusetts.; ^5^ VA South Central Mental Illness Research, Education, and Clinical Center, Houston, Texas.; ^6^ Baylor College of Medicine, Houston, Texas.; ^7^ VA Loma Linda Healthcare System, Loma Linda, California.; ^8^ Minneapolis VA Health Care System, Minneapolis, Minnesota.; ^9^ Southern Arizona VA Health Care System, Tucson, Arizona.; ^10^ East Tennessee State University and Mountain Home VAMC, Johnson City, Tennessee.; ^11^ University of Houston, Houston, Texas.; ^12^ Michael E. DeBakey VA Medical Center, Houston, Texas.; ^13^ VA Greater Los Angeles Healthcare System, Los Angeles, California.; ^14^ Edith Nourse Rogers Memorial Veterans Hospital, Bedford, Massachusetts.; ^15^ James J Peters VA Medical Center, Bronx, New York.; ^16^ Icahn School of Medicine at Mount Sinai, New York, New York.

**Keywords:** access to care, consultation, gender dysphoria, gender identity, gender transition, interdisciplinary, intersex, training, transgender, veteran

## Abstract

**Purpose:** The Veteran's Health Administration (VHA) has created a training program for interdisciplinary teams of providers on the unique treatment needs of transgender veterans. An overview of this program's structure and content is described along with an evaluation of each session and the program overall.

**Methods:** A specialty care team delivered 14 didactic courses supplemented with case consultation twice per month over the course of 7 months through video teleconferencing to 16 teams of learners. Each team, consisting of at least one mental health provider (e.g., social worker, psychologist, or psychiatrist) and one medical provider (e.g., physician, nurse, physician assistant, advanced practice nurse, or pharmacist), received training and consultation on transgender veteran care.

**Results:** In the first three waves of learners, 111 providers across a variety of disciplines attended the sessions and received training. Didactic topics included hormone therapy initiation and adjustments, primary care issues, advocacy within the system, and psychotherapy issues. Responses were provided to 39 veteran-specific consult questions to augment learning. Learners reported an increase in knowledge plus an increase in team cohesion and functioning. As a result, learners anticipated treating more transgender veterans in the future.

**Conclusion:** VHA providers are learning about the unique healthcare needs of transgender veterans and benefitting from the training opportunity offered through the Transgender Specialty Care Access Network–Extension of Community Healthcare Outcomes program. The success of this program in training interdisciplinary teams of providers suggests that it might serve as a model for other large healthcare systems. In addition, it provides a path forward for individual learners (both within VHA and in the community) who wish to increase their knowledge.

## Introduction

The Veteran's Health Administration (VHA) created a comprehensive national policy on transgender care in 2011 (now VHA Directive 2013-003).^1^ Based on analyses of diagnostic codes, the number of transgender veterans in VHA has been increasing since 2008 with a significant increase after 2011.^2^ Approximately 2600 unique transgender veterans used VHA services in 2013,^2^ although other studies find higher rates of VHA use,^3,4^ and additional transgender veterans may choose to receive care outside of VHA. Although increasing in number system-wide, the number of transgender veterans at any one facility is usually quite small.

Few VHA providers have training or experience in transgender veteran treatment.^5,6^ Specialized services that VHA providers are expected to deliver include gender identity counseling, evaluations for hormone therapy, prescribing hormones, primary care services, evaluations for, but not provision of, masculinizing/feminizing surgeries, postsurgical care, and support letters for name and gender marker change within the VHA system and externally. However, for VHA providers with little or no experience or training in this specialized care, the mandate to provide culturally competent transgender healthcare can be daunting.

The World Professional Association for Transgender Health (WPATH) has issued the most widely accepted standards of care (SOC).^7^ However, VHA is not required to follow the WPATH SOC or other external guidelines such as the Endocrine Society's guidelines for transgender care.^8^ This may create confusion for some VHA providers. Where guidance exists on transgender veteran care, it is often specific to a particular discipline, such as psychology,^9^ despite the fact that transgender care is inherently interdisciplinary. Thus, providers may be unsure what core competencies they should follow to treat transgender veterans.^10^

In part, to address this need, the Lesbian, Gay, Bisexual, and Transgender (LGBT) Program was established within the Office of Patient Care Services in VHA. Through this office, VHA has been delivering trainings on transgender veteran care in a variety of formats and depth of content. Given the complexities of care for transgender veterans, it was determined that a more detailed educational program was needed to train interdisciplinary teams of VHA providers in every region of the country. This strategy can better ensure clinically competent and culturally sensitive transgender care for veterans nationwide.

Based on the success of the VHA Specialty Care Access Network–Extension of Community Healthcare Outcomes (SCAN-ECHO) programs, which train providers in remote settings in medical subspecialty domains (e.g., diabetes), a national Transgender SCAN-ECHO program was developed and implemented to provide clinical consultation and education for interdisciplinary teams of providers.^11,12^ The development of VHA Transgender SCAN-ECHO is described in more detail elsewhere.^11^ The current article has two aims: (1) describe the curriculum of the Transgender SCAN-ECHO program as a minimum of core content for learners who treat transgender veterans and (2) report on the performance of Transgender SCAN-ECHO from the first three cohorts of learners.

## Methods

Cohorts of learners in the Transgender SCAN-ECHO program were taught by the specialty team during 14 one-hour videoconference sessions, meeting twice per month over the course of 7 months. Each session included a 15–20-min didactic presentation by an interdisciplinary specialty team, supplemented by case consultation for 30–45 min. In this way, instruction was provided on VHA policies and practices for each element of treatment.^13–14^ The goal of Transgender SCAN-ECHO was to train teams of VHA providers across the country to increase capacity to treat transgender veterans at their local facility and to serve as a resource for VHA providers in their region.

### Transgender SCAN-ECHO specialty team

An interdisciplinary team of VHA providers with experience in transgender veteran healthcare was identified throughout the VHA system. The team underwent a yearlong process of training to ensure a similar and thorough understanding of transgender veteran care across disciplines, facilitate team building, and develop the didactic content and consult template for SCAN-ECHO learners (see Kauth et al.^11^). The team comprised a psychologist, social worker, primary care physician, nurse, pharmacist, and endocrinologist. The identification of a highly motivated specialty team was pivotal to the success of the program. The team participated in twice monthly calls and a face-to-face workshop as part of the training. Training of the specialty team was undertaken by VHA clinical staff with at least a decade each of experience treating transgender veterans. One trainer was also a coauthor of the WPATH SOC,^7^ which the training content largely followed, although this training was tailored to VHA policy and practices and veteran-specific culture. All specialty team members were involved in development of the SCAN-ECHO curriculum (discussed below), led specific didactic trainings, and participated in case consultations.

### Transgender SCAN-ECHO learners

The interdisciplinary teams of learners were recruited from throughout VHA through dissemination of an overview of the program through established VA notification pathways (see Kauth et al.^11^). Teams were required to have at least one medical provider (e.g., physician, nurse, physician assistant, advanced practice nurse, or pharmacist) and one mental health provider (e.g., social worker, psychologist, or psychiatrist) willing to devote at least 14 h of time over 7 months to learn about transgender care. Additionally, teams needed to have access to the secure VHA videoconferencing equipment. Teams also agreed to obtain the veteran's consent and then submit case-specific consult questions through the electronic medical record at least 1 week in advance of the session to allow time for chart review. These cases were presented by the learner team during the SCAN-ECHO session and discussed in real time by the specialty team. Following the session, the specialty team summarized the interdisciplinary response in the veteran's medical record. In addition, learner teams were required to attend a minimum of 80% of sessions and complete post-tests for each session. Continuing education (CE) hours were offered with each session. Learners also completed an overall evaluation at the end of the SCAN-ECHO program.

Five interdisciplinary VHA learner teams participated in the first cohort (January through July 2014) and included Cincinnati, OH; Durham, NC; Miami, FL; North Chicago, IL; and Oklahoma City, OK. The composition of each team varied in number and discipline (Table 1). A total of 33 clinicians participated in the first cohort, representing nursing, pharmacy, physicians, physician's assistants, psychologists, and social workers. During the 7-month program, 10 consults were submitted for case consultation. The second and third cohorts of learners participated concurrently on alternating weeks from August 2014 through March 2015. Teams in the second and third cohorts included 11 VHA sites: Indianapolis, IN; Hines, IL; Baltimore, MD; Manhattan, NY; Salt Lake City, UT; Portland, OR; Little Rock, AR; Shreveport, LA; Oakland/Sacramento, CA; Las Vegas, NV; and Boise, ID. A total of 78 clinicians (Cohort 2=35; Cohort 3=43) completed training. Learners included nurses, nurse practitioners, pharmacists, psychologists, social workers, psychiatrists, physicians, physician assistants, dieticians, chaplains, and speech/language pathologists (Table 1). During the program, 29 consults (Cohort 2=16; Cohort 3=13) were submitted for consultation. A summary of the primary consult questions is listed in Table 2, although the majority of consults included more than one question.

**Table 1.** **Transgender SCAN-ECHO Learners by Site, Cohort, and Discipline**

	RN	NP	Pharmacist	Physician	PA	Psychologist	Psychiatrist	SW	Other	Total learners
Cohort 1										
Cincinnati	2	0	1	1	0	2	0	3	0	9
Durham	0	1	0	1	0	1	0	1	0	4
Miami	0	0	0	3	0	1	0	3	0	7
North Chicago	2	1	0	1	1	1	0	1	0	7
Oklahoma	1	0	1	3	0	1	0	0	0	6
Total per discipline	5	2	2	9	1	6	0	8	0	33
Cohort 2										
Baltimore	2	0	1	0	0	1	0	3	1	8
Hines	0	0	1	0	0	3	4	1	1	10
Indianapolis	1	0	2	2	1	1	0	0	0	7
Manhattan	1	0	0	2	0	1	0	0	0	4
Salt Lake City	2	0	0	1	0	0	0	2	1	6
Total per discipline	6	0	4	5	1	6	4	6	3	35
Cohort 3										
Boise	1	0	0	2	0	2	0	1	0	6
Las Vegas	0	0	1	1	0	0	0	0	0	2
Little Rock	0	0	0	3	0	3	0	3	0	9
North California	0	0	1	1	0	1	0	3	3	9
Portland	0	1	0	0	0	2	0	3	0	6
Shreveport	1	0	2	0	0	4	1	3	0	11
Total per discipline	2	1	4	7	0	12	1	13	3	43
Overall Total per discipline	13	3	10	21	2	24	5	27	6	111

Other; Chaplain, Registered Dietician, Speech Pathologist.

NP, nurse practitioner; PA, physician assistant; RN, registered nurse; SCAN-ECHO, Specialty Care Access Network–Extension of Community Healthcare Outcomes; SW, social worker.

**Table 2.** **Summary of Primary Consult Question**

Consult question	Cohort 1	Cohort 2	Cohort 3	Total
Advocacy within the system	0	2	2	4
Documentation/record changes	1	0	1	2
Primary care	3	1	2	6
Evaluation for hormone readiness	0	1	1	2
Hormone therapy initiation	0	1	1	2
Hormone therapy adjustment/reevaluation	3	2	3	8
Evaluation for gender-confirming surgery readiness	1	2	0	3
Psychotherapy for gender dysphoria	0	2	0	2
Psychotherapy for other problem areas	1	1	1	3
Social resources	1	0	0	1
Presurgical care	0	1	0	1
Postsurgical care	0	0	1	1
Other	0	3	1	4
Total	10	16	13	39

Other was classified when the consult listed five or more questions in the same consult (*N*=3) or when questions occurred regarding VA policy that prohibited surgical interventions (*N*=1).

### Transgender SCAN-ECHO curriculum

The specialty team and LGBT Program administrators developed 14 brief (15–20 min) didactics on transgender care as the curriculum. Didactics were modeled after the specialty team's own training and determined to be essential core content. Post-training questions were also created for each didactic to ensure that learners understood the information. Learners were required to achieve an 80% passing score on each post-test to receive CE hours.

### Overview of the didactics

Didactic content is described below in the order that they were presented.

(1) Transgender 101: a basic overview was provided on transgender veteran care, including the VHA Directive 2013-003, WPATH SOC, and gender identity terms. Considering the social stigma and explicit bias in healthcare that transgender veterans often experience,^5,6,15,16^ this session focused on the importance of reducing stigma and promoting a supportive healthcare environment. Topics included the VHA patient nondiscrimination policy and the requirement that veterans must be treated based upon their self-identified gender (irrespective of appearance or surgical status) in face-to-face conversations, room assignments, bathroom use, and in documentation. VHA has a zero tolerance policy for discrimination.
(2) Enhancing provider and patient communication: the quality of provider–patient communication has been shown to be positively correlated with patient satisfaction, information recall, compliance with therapeutic regime, and appointment keeping.^17,18^ Emphasis was placed on teaching providers essential communication techniques (e.g., nonjudgmental attitude, positive regard, and empathetic communication) to establish and maintain positive rapport with patients. Creating a positive fear-free alliance facilitates more comfortable and truthful discussions, thereby improving diagnosis and treatment.^19^
(3) Mental health evaluation for hormone therapy: both WPATH SOC and VHA policy recommend a mental health evaluation before initiating hormone therapy to establish (1) a gender dysphoria^20^ diagnosis and (2) patient's capacity to make an informed medical decision. Evaluations are intended to maximize treatment success by ensuring the patient is adequately informed and prepared to start hormones. Instruction was provided on how to diagnose gender dysphoria and differential diagnoses and how to assess the decision-making capacity, as well as how to discuss the veteran's transition-related goals and understanding of the risks, benefits, limits, and permanent effects of hormone interventions (e.g., infertility). Learners were also encouraged to evaluate past and current mental health concerns and treatment goals. Finally, learners were instructed on how to make clear behavioral recommendations to enhance a veteran's success in hormone therapy and gender transition. This didactic stressed the importance of advocacy on behalf of the veteran rather than gatekeeping.
(4) Transitioning: stages of transgender identity development (using Lev's model)^21^ and therapeutic tasks for each stage were described, recognizing that for many, transitioning is not a set linear process. For example, awareness of gender incongruity occurs when an individual becomes aware of feelings that they might identify as transgender. The therapeutic tasks include normalizing this experience, creating a safe space to explore gender, and consideration of various options of gender expression. However, people may follow various transition pathways. The focus was on identity integration, which is multidimensional and individual.
(5) Medical aspects of transgender care: general health management and health risks and benefits associated with the use of hormones were reviewed. The importance of routine health screenings was reinforced given that automatic clinical reminders tied to birth sex are altered after a veteran changes the sex marker in their electronic medical record, with important screenings no longer being required. The didactic emphasized the ongoing assessment of health risks such as endocrine-dependent cancers from estrogen use and vascular events associated with both estrogen and testosterone. This didactic served as the foundation for conducting a personalized medical risk assessment balanced with a discussion about the realistic changes that can be expected with hormone therapy (when relevant).
(6) Being a good consultant: upon completion of this program, learners were expected to be a local resource on transgender care for providers in their VHA facility and regionally. This didactic compared and contrasted the direct patient care role with the consultant role. Learners reviewed strategies for creating supportive consulting relationships that encourage open ongoing communication and reviewed important VHA resources for information (including E-consultation) and other available trainings (online webinars). Learners were taught to validate the need for consultation, acknowledge the complexity of transgender care, give corrective information, and avoid common pitfalls.
(7) Medical assessment for feminizing hormones: important medical risks and benefits specific to the use of feminizing hormones were addressed. The medical assessment focused on managing coexisting medical conditions such as smoking, liver disease, hyperlipidemia, diabetes, cardiovascular disease, including hypertension and history of thrombosis, migraines, cholelithiasis, or cancer history. Any of these conditions can be adversely affected by estrogen and should be well managed before estrogen is initiated. This didactic also described the use of medications that block endogenous androgens or lower androgen levels. The expected physical changes that occur with estrogen therapy in natal males were described for each body system to promote realistic expectations and raise awareness about possible medical complications.
(8) Prescribing feminizing hormones: information was provided on the various medication choices recommended by the Endocrine Society and the VHA Pharmacy guidelines. The therapeutic rationale for each medication was described as well as the expected benefits and risks. Emphasis was placed on an individualized patient-centered approach. The recommended strategy involved starting with antiandrogens (e.g., spironolactone, finasteride), then adding an estradiol preparation. Required clinical and laboratory parameters for initiation and follow-up were described with an emphasis on medication compliance and safety. Medications such as ethyl estradiol, conjugated equine estrogen, and progesterone preparations were not recommended.
(9) Medical assessment for masculinizing hormones: this session focused on how to conduct a medical risk and benefit assessment before initiating hormones for transgender men. Recommendations included assessment of coexisting medical conditions such as smoking, liver disease, hyperlipidemia, diabetes, cardiovascular disease, including history of thrombosis, severe renal disease, pregnancy or breast feeding, and cancer history. Any of these conditions can be adversely affected by testosterone and should be well managed before testosterone is initiated. Providers were encouraged to confirm patients' understanding of the risks, benefits, limits, and permanent effects of testosterone therapy, including the development of facial hair, deepening of the voice, and infertility.
(10) Prescribing masculinizing hormones: information on medication choices supported by the Endocrine Society and the current VHA Pharmacy guidelines was provided. The primary medication used for masculinization is testosterone with or without additional agents (e.g., progestins in the short term to mitigate menses). The therapeutic rationale for each medication and respective dosages was described. This didactic emphasized an individualized patient-centered approach, guided by the expected benefits and risks for each medication. The specialty team described their approach in medication choice with various testosterone preparations (intramuscular, transdermal patch, or gel) and progestin therapy based on medical risks and patient preference. Careful clinical and laboratory monitoring was emphasized, along with medication compliance and safety (including contact precautions with some formulations). Learners were advised to aim for typical physiological levels of testosterone for natal males and avoid supraphysiological levels. Learners were also encouraged to monitor preexisting conditions for change and check for the onset of new conditions secondary to the treatment (e.g., acne, male pattern baldness).
(11) Writing support letters for transgender veterans: this didactic covered various kinds of letters provided to transgender veterans to support their transition-related goals. For example, a carry letter is used to inform authorities that the individual is undergoing treatment and may appear as a gender different than expected, and/or markers may be inconsistent across documents, that is, the individual's appearance may not match the name/sex listed on identity documents such as a driver's license or state-issued identification card. Key elements of effective support letters for hormone therapy and transition-related surgeries outside of VHA, as well as letters supporting change of gender in VHA records, driver's licenses, and passports, were also discussed.
(12) Varieties of masculinizing surgeries: two sessions were devoted to gender-confirming surgeries (GCS) or sex reassignment surgeries (SRS). VHA clinicians may be involved in the presurgical evaluation and referral process, as well as post-GCS surgical care. These didactics described the presurgical evaluation and referral process and potential postsurgical and medical issues. VHA must provide treatment (or pay for non-VHA care) for complications of GCS procedures performed by non-VHA surgeons, making an understanding of these procedures and potential complications essential. Emphasis was placed on the medical necessity of such surgical treatments for individuals with gender dysphoria and the irreversibility of these procedures. Reproductive decision-making before GCS was also discussed. This didactic included descriptions and photographs of common outcomes experienced by patients undergoing masculinizing procedures, such as chest reconstruction and genital surgeries (e.g., hysterectomy, metoidioplasty, or phalloplasty).
(13) Varieties of feminizing surgeries: feminizing surgical interventions were covered focusing on many of the same issues addressed for masculinizing surgeries. The WPATH criteria were presented for each potential surgery (e.g., breast augmentation, vaginoplasty, and penectomy without vaginoplasty). For example, the criteria for vaginoplasty include persistent, well-documented gender dysphoria; capacity to make a fully informed decision and consent for treatment; age 18; significant medical or mental health concerns are reasonably well controlled; 12 continuous months of hormone therapy (unless the patient has a medical contraindication or is otherwise unable or unwilling to take hormones); 12 continuous months of living in a gender role that is congruent with their gender identity; and two referral letters from mental health providers who have been trained to work with gender dysphoric adults. This didactic noted that the specific requirements of surgeons vary and care should be coordinated.
(14) Introduction to disorders of sex development (DSD): a historical context and overview of theories and practices of initial gender assignment of individuals with DSD were offered. Content covered the distinctions between the terms *DSD*, *somatic intersex condition*, and *intersex identity*; the role of intersex advocacy groups in the evolution of current treatment practices; ethical considerations raised by irreversible SRS performed on infants; the importance of age-appropriate disclosure of DSD to affected individuals; common experiences and challenges faced by individuals with DSD and their families; gender identity in individuals with DSD; differences across DSD syndromes in rates of gender dysphoria and gender transition; and changes in DSM-5^20^ that allow individuals with DSD to be diagnosed with gender dysphoria.

## Measures

### Evaluation of sessions

Each learning session was evaluated in four ways. First, following each session, learners took a post-test consisting of 10 questions with a score of 80% or higher needed for a passing score. Second, learners evaluated the session by responding to five questions rated on a 0–5 Likert scale (0=not applicable, 1=strongly disagree, 2=disagree, 3=neutral, 4=agree, 5=strongly agree): (1) *Overall, I was satisfied with this learning activity*; (2) *The learning activities were effective in helping me learn the content*; (3) *I learned new knowledge and skills*; (4) *The scope of the learning activity was appropriate to my professional needs*; and (5) *I will be able to apply the knowledge and skills learned to improve my job performance.* Third, learners rated their agreement with meeting the objectives for each session. Finally, learners described what they considered to be the most useful and also least useful elements of the training using free text.

### Evaluation of the overall program

At the conclusion of the SCAN-ECHO program, all learners received a SurveyMonkey link to a brief anonymous evaluation of the overall program. Evaluation questions were developed by the LGBT Program administrators and specialty team and consisted of Likert-scale and open-text items. Learners gave overall ratings of several facets of the program and rated specific elements of the training (e.g., didactic learning, getting consultation, and listening to other consultations). Learners were asked to rate their confidence in treating transgender veterans (0–100%) before participating in SCAN-ECHO and after SCAN-ECHO and their expectations to treat the same, or less, or more transgender veterans post-training. Finally, learners were asked about the effect of the training on the treatment team dynamic and which didactics they found most and least useful, as well as suggestions for what topics should be added to the series.

## Results

Successful team attendance was set at 12 of 14 (80%) sessions. Only one of the 16 teams failed to meet this threshold at 79% attendance. Data for all teams were included in the analyses.

### Post-test data

Only a subset of learners completed the post-tests and received CE hours. Participation in post-tests varied from 11 to 57 learners, with an average of 22 participants completing post-test questions. Across the didactics, 93% of learners received a passing score of 80% or better on the 10-item quizzes (Table 3).

**Table 3.** **Post-Test Scores for Each Didactic Session**

Didactic	No. took test	No. passed	Passed (%)
Transgender 101	57	57	100
Enhancing provider and patient communication	30	29	97
Mental health evaluation for hormone therapy	32	31	97
Transitioning	24	22	92
Medical aspects of transgender care	17	16	94
Being a good consultant	23	22	96
Medical assessment for feminizing hormones	25	21	84
Prescribing feminizing hormones	17	16	94
Medical assessment for masculinizing hormones	16	16	100
Prescribing masculinizing hormones	11	10	91
Writing support letters for transgender veterans	14	14	100
Varieties of masculinizing surgeries	12	11	92
Varieties of feminizing surgeries	11	9	82
Introduction to DSD	20	19	80

DSD, disorders of sex development.

### Evaluation of learning session

Learners were asked to rate the teaching performance of the specialty team, inclusive of the didactic and case consultation. Summary responses are provided in Table 4. Satisfaction was high, with an average rating of 4.28 (on a 0–5 Likert scale) across all sessions. Similarly, effectiveness (average=4.31), gaining new knowledge and skills (average=4.23), meeting professional needs (average=4.23), and being able to apply their new knowledge and skills (average=4.18) were all highly rated across sessions.

**Table 4.** **Evaluation of Each Didactic**

Didactic	Overall satisfaction (SD)	Effective tools (SD)	Learned (SD)	Scope (SD)	Will apply (SD)	Training goals met (%)
Transgender 101	4.46 (0.60)	4.33 (0.51)	4.12 (0.73)	4.34 (0.61)	4.40 (0.63)	93
Enhancing provider and patient communication	4.22 (0.63)	4.26 (0.61)	4.07 (0.86)	4.11 (0.86)	4.30 (0.71)	71
Mental health evaluation for hormone therapy	4.38 (0.61)	4.25 (0.87)	4.31 (0.79)	4.41 (0.62)	4.38 (0.61)	92
Transitioning	4.38 (0.71)	4.42 (0.59)	4.38 (0.58)	4.43 (0.66)	4.43 (0.66)	86
Medical aspects of transgender care	4.44 (0.50)	4.30 (0.60)	4.44 (0.50)	4.44 (0.50)	4.22 (0.60)	87
Being a good consultant	4.36 (0.64)	4.30 (0.64)	4.27 (0.62)	4.32 (0.62)	4.27 (0.62)	91
Medical assessment for feminizing hormones	4.29 (0.63)	4.30 (0.64)	4.19 (0.85)	4.20 (0.81)	4.19 (0.59)	80
Prescribing feminizing hormones	4.25 (0.66)	4.31 (0.61)	4.19 (0.63)	4.25 (0.66)	4.19 (0.63)	81
Medical assessment for masculinizing hormones	3.94 (0.66)	3.93 (0.59)	4.13 (0.70)	3.88 (0.70)	3.88 (0.68)	80
Prescribing masculinizing hormones	3.90 (0.30)	4.13 (0.33)	3.90 (0.54)	4.00 (0.45)	4.00 (0.63)	79
Writing support letters for transgender veterans	4.31 (0.72)	4.45 (0.66)	4.15 (0.66)	4.15 (0.66)	4.31 (0.61)	87
Varieties of masculinizing surgeries	4.18 (0.72)	4.33 (0.67)	4.36 (0.64)	4.18 (0.72)	3.91 (0.51)	77
Varieties of feminizing surgeries	4.44 (0.68)	4.75 (0.43)	4.44 (0.68)	4.33 (0.67)	4.22 (0.63)	80
Introduction to DSD	4.41 (0.60)	4.21 (0.41)	4.29 (0.57)	4.12 (0.47)	3.88 (0.32)	81

0=not applicable; 1=strongly disagree, 2=disagree, 3=neutral, 4=agree, 5=strongly agree.

Across sessions, 83% of learners reported that the objectives of the session were met, irrespective of whether the content was specific to their discipline. Psychologists and social workers tended to rate medically driven sessions (e.g., prescribing hormones) at lower levels for meeting the stated objectives. Comments from the least useful content question reflected this trend. For example, “This presentation is geared toward MD or prescribing professionals. Less helpful (although necessary information) for mental health professionals.” Other common responses for least useful content focused on technical issues such as “Technical difficulties prevented slides from showing on our screen, glad that we have access to the slides to be able to review content.” A frequent response for most helpful content was the case consultation and discussion: “The opportunity to have free discussion of problematic issues” and “The case presentation and discussion by various disciplines.”

### Evaluation of the overall program

Of the 111 learners, 73 completed an overall evaluation of the program (66% response rate). Learners rated all of the elements of the program to be useful. Eighty-eight percent found *Knowing who to contact in the future* to be the most helpful aspect of the program, followed by *Listening to other cases being discussed* (87.5%) and *Resources on the SharePoint* (87%) (an online repository of reference material). Most learners also rated the didactics and receiving consultation components of the program as helpful (79% and 84%, respectively). Overall, confidence in treating transgender veterans (0–100%) improved for 92% of learners, increasing from 56.4% before SCAN-ECHO participation to 80.2% after SCAN-ECHO. Most learners (63%) also expected to treat more transgender veterans in the future, while 37% expected to treat about the same number. Team functioning improved with 77% of learners reporting that there was no team before this training and “this training helped us to discuss our cases.” Learners frequently cited improved knowledge of treatment, better communication among team members, and increased empathy and understanding of transgender veterans as positive changes within the team as a result of participation in SCAN-ECHO.

## Discussion

This article presents an overview of the content development and performance of the first national interdisciplinary training program for the treatment of transgender veterans. In the first three cohorts of learners, 111 interdisciplinary VHA providers were trained. All didactic sessions performed well, with learners from a variety of disciplines reporting that they were satisfied. Overall, learners rated the didactics as effective and reported acquiring new knowledge and skills. Veteran-specific case consultation was offered both in session and in the veteran's electronic medical record for 39 treatment questions.

Feedback from learners indicated that the Transgender SCAN-ECHO program as a whole was more important than the didactics alone. Key strengths of the program were facilitating connections among providers, both at the local level by enhanced team functioning and at the national level, with participants rating “Knowing who to contact” as an important benefit. It should be noted that an additional national VHA program of transgender E-consultation was also promoted throughout the SCAN-ECHO program. Thus, learners were aware that they could continue to get consultation on specific questions after completing Transgender SCAN-ECHO. Importantly, 77% of learners reported that team functioning had improved as a result of participating in the program, and 92% felt that their confidence in treating transgender veterans had improved. Indeed, most learners expected to treat more transgender veterans after participation.

In addition to learners improving, the SCAN-ECHO program improved as a result of feedback. Specifically, after reviewing feedback from these cohorts, the specialty team crafted a didactic on gender nonconforming identities, which replaced the didactic on effective communication. In part, the decision to replace the didactic on effective communication was made because the content was general and not specific to transgender healthcare. Instead, the material on communication was interwoven throughout the other didactics for the new cohorts of learners. The new didactic included terms and concepts to help providers work with veterans who identify with nonbinary genders. In addition, the effective consultant didactic was moved to the end of the series. A continual evaluation process allows the curriculum to evolve to meet the needs of learners.

A limitation of the work described above is that at the time of this report, training participation was somewhat limited geographically. In particular, coverage in the central and northern Midwest was sparse. Training through this pilot program will continue through fiscal year 2016. By the end of the pilot period (September 2016), a decision will be made regarding additional funding. By that date, there will be an interdisciplinary team trained in each Veteran Integrated Service Network (VISN) for VHA, including one in Alaska and one in Hawaii. Moreover, it has been demonstrated that both VHA hospitals and also community-based outpatient clinics have benefited from this training. However, this is viewed as a minimum in being able to provide informed competent care to transgender veterans at every facility. Ongoing transgender SCAN ECHO training could address the gaps in training and experience across large and small facilities, as well as rural and urban locales. Moreover, continuation of the SCAN ECHO program would assist in addressing provider turnover and allow training to be disseminated to new staff without the initial costly resource investment to reestablish the program (see Kauth et al.^11^). If VA policy on transition-related surgeries were to change (which are not currently provided), the transgender SCAN-ECHO program would be well positioned to respond to the need for education and training in the field on application of these changes. It is also important to note that the VHA transgender E-consultation program could also assist providers who require patient-specific consultation, irrespective of SCAN ECHO training. Currently, there are several teams of providers who are interested in participating in transgender SCAN ECHO if new cohorts of learners are approved. This overview of the content of the Transgender SCAN-ECHO program provides readers within and outside VHA with a clear description of training material delivered to providers. This information is relevant to VHA providers who may be interested in becoming SCAN-ECHO participants and for providers in the community who treat veterans.^22^ Indeed, during one of the training cohorts, an interdisciplinary team of providers from the Department of Defense (DoD) audited the training. DoD leaders also met with the VHA LGBT Program to discuss how VHA has implemented their transgender directive and provided training to clinical staff as DoD considers how to offer transgender service members quality and respectful care. Our aim was to describe the essential components of quality care for transgender veterans. We encourage readers to seek out training in these specific areas to improve their knowledge and skills and provide the best care possible to transgender patients. In addition, in combination with our previously published report,^11^ it provides a clear framework for other healthcare organizations that wish to improve their ability to treat transgender patients. The VHA Transgender SCAN-ECHO program offers a solution to increase provider knowledge and thereby improve direct services offered to transgender patients.

## Abbreviations Used

CE: continuing education
DoD: Department of Defense
DSD: disorders of sex development
GCS: gender-confirming surgeries
SCAN-ECHO: Specialty Care Access Network–Extension of Community Healthcare Outcomes
SOC: standards of care
SRS: sex reassignment surgeries
VHA: Veteran's Health Administration
WPATH: World Professional Association for Transgender Health
